# Investigating the missing data mechanism in quality of life outcomes: a comparison of approaches

**DOI:** 10.1186/1477-7525-7-57

**Published:** 2009-06-22

**Authors:** Shona Fielding, Peter M Fayers, Craig R Ramsay

**Affiliations:** 1 Section of Population Health, University of Aberdeen, UK; 2 Department of Cancer Research and Molecular Medicine, Faculty of Medicine, Norwegian University of Science and Technology, Trondheim, Norway; 3 Health Services Research Unit, University of Aberdeen, UK

## Abstract

**Background:**

Missing data is classified as missing completely at random (MCAR), missing at random (MAR) or missing not at random (MNAR). Knowing the mechanism is useful in identifying the most appropriate analysis. The first aim was to compare different methods for identifying this missing data mechanism to determine if they gave consistent conclusions. Secondly, to investigate whether the reminder-response data can be utilised to help identify the missing data mechanism.

**Methods:**

Five clinical trial datasets that employed a reminder system at follow-up were used. Some quality of life questionnaires were initially missing, but later recovered through reminders. Four methods of determining the missing data mechanism were applied. Two response data scenarios were considered. Firstly, immediate data only; secondly, all observed responses (including reminder-response).

**Results:**

In three of five trials the hypothesis tests found evidence against the MCAR assumption. Logistic regression suggested MAR, but was able to use the reminder-collected data to highlight potential MNAR data in two trials.

**Conclusion:**

The four methods were consistent in determining the missingness mechanism. One hypothesis test was preferred as it is applicable with intermittent missingness. Some inconsistencies between the two data scenarios were found. Ignoring the reminder data could potentially give a distorted view of the missingness mechanism. Utilising reminder data allowed the possibility of MNAR to be considered.

## Background

Missing data are a major issue during the analysis of any study. The absence of data can be informative, and should not be disregarded; ignoring the pattern of missingness may bias the results obtained. In particular, for health-related quality of life (QoL) outcomes, the fact that data are missing may be informative. Patients who feel unwell are perhaps likely to be less able to complete and return questionnaires.

Patterns of missingness are described as either monotone (terminal), intermittent or mixed. Monotone missingness occurs when data are available at every assessment until a time the patient drops out and provides no further assessments. Intermittent missingness occurs if there is a missing observation in between observed assessments. A mixed pattern occurs when a period of intermittent missingness is followed by monotone missingness. The three mechanisms of missing data are missing completely at random (MCAR), missing at random (MAR) and missing not at random (MNAR) [1]. Determining the mechanism helps to identify the most appropriate analysis method. Complete-case analysis (excluding patients who have incomplete data) will only be unbiased (although not optimal) if the data are MCAR. Under MAR, available case analysis such as mixed effects models can be used whereas for MNAR data fewer, more sophisticated methods are appropriate [2].

The Centre for Healthcare Randomised Trials at the University of Aberdeen routinely employs a reminder system when administering follow-up questionnaires. When a patient does not respond within two weeks of the initial mailing, a reminder questionnaire is sent and a second, two weeks later when required. At each assessment there are three types of responder: immediate-responders (no reminder necessary), reminder-responders (responded following one or more reminders), and non-responders. We aim to determine if the reminder-response data can be utilised to identify the non-response mechanism. We compare the missingness mechanism when the reminder-response data is included (that is using all available data) and excluded (as they would be in those clinical trials that do not employ a reminder system). Four different methods to identify the missingness mechanism were applied and contrasted.

## Methods

### Datasets

Datasets from five clinical trials that administered the EuroQoL EQ5D [3] instrument were used. The EQ5D is a generic QoL questionnaire, with five questions covering: mobility, self-care, usual activities, pain/discomfort and anxiety/depression. Each question has a three-category response scale, with a single index generated for all health states, using the EuroQoL UK population tariff. This generates 3 × 3 × 3 × 3 × 3 = 243 unique values ranging from -0.59 (worst QoL) to 1 (best QoL). The EQ5D score is usually treated as a continuous variable. The five trials are:

1. REFLUX (N = 357) – evaluating the clinical- and cost-effectiveness of early laparoscopic surgery compared with continued medical management amongst people with gastro-oesophageal reflux disease. QoL data was collected at baseline, three and twelve months after surgery, and at equivalent times for those medically managed [4].

2. MAVIS (N = 910) – RCT of multi-vitamin and mineral supplementation in persons aged 65 and over, to reduce infection rates and antibiotic usage. QoL data was collected at baseline, six and twelve months follow-up [5].

3. RECORD (N = 5292) – a placebo-controlled trial of daily oral vitamin D and calcium in the secondary prevention of osteoporosis-related fractures in older people. QoL was assessed at four months (baseline) and then yearly up to four years [6].

4. KAT – overlapping trials measuring clinical and cost effectiveness of different types of knee replacement. The comparison presented evaluates the benefits of patella resurfacing during knee replacement (N = 1517). QoL was measured at baseline, three months and annually after the operation [7].

5. PRISM (N = 1324) – evaluating the clinical- and cost-effectiveness of symptomatic versus intensive biphosphonate therapy for the management of Paget's disease. QoL was assessed at baseline and then annually [8].

Each dataset contained a proportion of patients with complete data or a monotone, intermittent or mixed missing data pattern.

### Mechanisms of missing data

The missing data 'mechanism' relates to the underlying reason why the data are missing. Rubin [1] presents the standard definition of the missing data mechanism which can be classified as MCAR, MAR or MNAR (see Appendix for formal definition). In summary, MCAR depends on observed covariates, but not on the observed or unobserved outcomes. The MAR mechanism depends on the observed outcomes and perhaps covariates, but not further on unobserved measurements. MNAR does depend on unobserved measurements, perhaps in addition to covariates and/or observed outcomes [9]. MCAR and MAR are often referred to as ignorable – that is if a dropout process is random then unbiased estimates can be obtained from likelihood-based estimation [2,10]. MNAR is non-ignorable, because to do so would lead to biased results.

In the context of QoL, MCAR occurs if the missingness has nothing to do with QoL status. For example, the form may be missing because it got lost in the post. MCAR includes 'covariate dependent missingness' – for example, if missingness varies between age groups, but within each age group, missingness is MCAR. When missingness is related to the observed QoL scores, we have MAR data. MNAR describes missingness that is related to unobserved QoL. An example would be missing values arising because severely ill patients felt too weak to complete questionnaires.

### Methods for determining the mechanism of missingness

There are a number of hypothesis tests that can be carried out to test for MCAR. Little [11] developed a test based on means under the different missing data patterns. Listing and Schlittgen also proposed a test based on means [12] and secondly a non-parametric procedure which combines several Wilcoxon rank sum tests [13]. Schmitz and Franz discussed a non-parametric version of the first Listing and Schlittgen test [14]. Diggle [15] used an approach which tests whether the subset about to dropout are a random sample of the whole population. Ridout [16] adopted a similar approach to Diggle, by utilising logistic regression. Fairclough [2] detailed a logistic regression procedure subtly different from that of Ridout.

The missing data patterns displayed by the example datasets are a mixture of monotone, intermittent and mixed. Of the hypothesis tests described, only Little's test can be applied to datasets containing intermittent and mixed patterns in addition to monotone patterns. The remaining hypothesis tests are restricted to monotone missingness. Therefore, Little's test [11] was chosen to be applied and despite requiring monotone missingness Listing and Schlittgen's parametric test [12] was chosen as a comparison. Both Ridout and Fairclough logistic regression were employed.

Little's test [11] and the Listing and Schlittgen test [12] provide a global view of the missingness mechanism. Fairclough's method [2] is similar to that of Ridout [16] but in Ridout's approach the indicator of missingness is between responders at a given assessment who continue in the study and those who do not. Fairclough's [2] missingness indicator distinguishes between responders and non-responders at each assessment. No restriction to the data is required for either logistic regression procedure. The mathematical details of these methods are found in the Appendix but are now described in non-technical language.

#### Little's test of MCAR

This test is based on the premise that under MCAR at each assessment the calculated means of the observed data should be the same irrespective of the pattern of missingness [11]. The null hypothesis is that the data are MCAR. If the data are not MCAR, the mean scores at each assessment will vary across the patterns.

#### Listing and Schlittgen (LS) test: to determine if dropouts are missed at random

Listing and Schlittgen [12] proposed a test (denoted the 'LS test') to determine if 'dropouts' occurred at random. This test requires a monotone missing data pattern and the null hypothesis is that the dropouts are missed at random. At each assessment a test is based on the difference in the mean of the values of the individuals who continue to stay in the study and the mean for those individuals who drop out after this time. The test statistic combines the weighted differences of the means of dropouts and non-dropouts at the different assessments (see Appendix). For the non-dropouts only the patients providing all assessments are used. This ensures that a possible continuing slow change in the means of later dropouts does not mask the differences of mean values by moving the mean of the non-dropouts into the direction of the mean of the dropouts.

#### Ridout's logistic regression method

Diggle [15] proposed a method of testing the hypothesis that dropouts occur at random within treatment groups against the alternative hypothesis that their occurrence is related to a particular covariate. Following this, Ridout proposed a comparable test for random dropouts in repeated measurement data using logistic regression [16]. At each assessment, one identifies the set of patients for whom assessment is available at that point and then identifies the subset for which this is the final assessment before they drop out of the study. The test for MCAR, tests the assumption that scores from the subset of subsequent dropouts are a random sample from all those providing assessment. The response variable is 'dropout or not at a particular assessment' in the standard logistic regression model [17]. It is possible under MCAR that dropout may depend on fixed covariates (covariate-dependent dropout).

#### Fairclough's logistic regression method

Fairclough outlined an approach to identify the missingness mechanism using logistic regression [2]. The first step is to identify any variables within the dataset that are associated with the indicator of missingness (response or not at a particular assessment). These could include demographic variables or other treatment related variables. A logistic model can be created from the significant candidate variables, using a stepwise procedure. Differences between MCAR and MAR can be assessed by examining the association of missing data with observed QoL scores, using logistic regression. To confirm that missingness depends on observed data after adjusting for the dependence on any covariates, the covariates are forced into the model and the observed QoL is tested for inclusion. If the observed QoL score is significant in the model predicting missingness then there is evidence of MAR data.

#### Comparison of immediate and reminder responders using Fairclough's method

By restricting the dataset to responders only and regarding the reminder-response as missing, Fairclough's logistic regression approach can be used to determine whether reminder-data are MNAR rather than MCAR or MAR. If the current QoL score is significant in the logistic model having adjusted for covariates and previously observed QoL, then there is evidence of possible MNAR data. This conclusion is only possible because we are using all responder data and the true value of the data which we are regarding as missing (in the indicator variable) is known.

#### Overview

To undertake the LS test restricted trial datasets using only those patients with a monotone missingness pattern were created. The four methods to determine the missingness mechanism were applied. Scenario one contains the immediate response data versus the missing data (reminder-response or actual non-responders). Scenario two includes response data (immediate and reminder responders) and investigates the mechanism behind non-response.

In addition, a subset of data which included only responders at each assessment was created. The responses received after reminders were set to missing and the mechanism behind reminder-response investigated. Fairclough logistic regression was used to determine whether the current score was a predictor of reminder-response, suggesting MNAR. With the rationale that reminder-responders are perhaps closer to the non-responders, if reminder-response is MNAR it implies that non-response is likely MNAR. Previous QoL is defined to be the last known QoL score. All analysis was undertaken in STATA/SE version 10.1 for Windows.

## Results

Table 1 shows the proportions of responders in each response category. MAVIS had an excellent response rate to the initial mailing, while REFLUX showed the poorest initial response rate. The reminder system generated a significant amount of data producing an overall response rate of 86% at three months and 89% at 12 months. RECORD showed the poorest overall response rate (22%–35% non-responders). The reminder system did generate about a quarter of all responses.

**Table 1:** Percentage of each type of responder in each trial

		Type of responder		
Trial	Assessment	Immediate	Reminder	Non-responder
REFLUX (N = 357)	3 months	39	47	14
	12 months	38	51	11
MAVIS (N = 910)	6 months	91	4	5
	12 months	81	11	8
RECORD (N = 5292)	4 months	58	20	22
	12 months	54	17	29
	24 months	51	14	35
KAT (N = 2356)	3 months	79	9	12
	1 year	74	13	13
	2 years	69	15	16
PRISM (N = 1324)	1 year	85	6	9
	2 years	63	14	23

Table 2 displays the baseline QoL scores split by responder type at the first follow up. In each of the five trials, the participants who responded immediately at first follow-up had better baseline QoL scores than those who were reminder-responders or non-responders. This pattern was particularly evident in REFLUX, MAVIS and RECORD. This suggests those patients who were displaying poorer baseline QoL were more likely to be a reminder-responders or non-responder at follow up, indicating a MAR mechanism. The four methods to determine the mechanism of missingness were used to confirm this hypothesis. Scenario one utilised the immediate responses and regarded reminder responders along with the true non-responders as missing. Scenario two included the reminder-response values in the responder set and missing data was only that arising from non-response.

**Table 2:** Baseline QoL scores split by responder type at first follow-up

		Immediate responders	Reminder responders	Non-responders
		Mean (SD)	Mean (SD)	Mean (SD)
REFLUX	EQ5D	0.75 (0.21)	0.70 (0.25)	0.70 (0.23)
(3 m)	Physical summary	45.2 (9.5)	44.9 (9.5)	45.5 (9.0)
	Mental Summary	47.3 (11.2)	44.5 (11.3)	42.1 (14.7)
	RQLS	66.8 (25.0)	64.3 (24.0)	64.0 (24.2)

MAVIS	EQ5D	0.77 (0.21)	0.73 (0.23)	0.70 (0.23)
(6 M)	Physical summary	43.6 (11.0)	40.9 (10.4)	40.0 (11.0)
	Mental Summary	53.9 (8.6)	51.7 (9.9)	52.2 (9.1)

RECORD	EQ5D	0.74 (0.23)	0.69 (0.25)	0.66 (0.29)
(12 m)	Physical summary	41.7 (10.7)	40.0 (11.1)	38.6 (11.8)
	Mental Summary	51.7 (9.9)	48.8 (10.3)	47.3 (11.4)

KAT	EQ5D	0.39 (0.31)	0.34 (0.31)	0.35 (0.32)
(3 m)	Physical summary	31.1 (8.2)	30.0 (8.7)	31.5 (8.3)
	Mental Summary	50.1 (11.4)	50.2 (11.8)	47.1 (12.0)
	Oxford Knee Score	18.2 (7.5)	17.0 (7.6)	17.5 (8.2)

PRISM	EQ5D	0.59 (0.30)	0.63 (0.27)	0.43 (0.34)
(12 m)	Physical summary	36.5 (11.4)	37.4 (10.9)	33.2 (10.0)
	Mental Summary	48.9 (11.8)	48.0 (11.8)	46.8 (12.1)
	Arthritis Index	36.1 (12.7)	36.1 (12.6)	31.9 (11.0)

### Hypothesis tests for mechanism of missingness

Table 3 shows the results of Little's hypothesis test of MCAR. In general there was evidence against MCAR, except for the MAVIS trial in scenario one and the PRISM trial in scenario two, where missingness was MCAR (covariate-dependent). The mechanism was consistent between these two scenarios except for the two cases above. In MAVIS scenario one was found to be MCAR while scenario two was not MCAR. Conversely in PRISM scenario one was not MCAR while there was no evidence against MCAR for scenario two.

**Table 3:** Results of Little's test

	Scenario 1		Scenario 2	
LITTLES TEST	Test Statistic (p-value)	MCAR?	Test Statistic (p-value)	MCAR?

REFLUX	18.6 (p = 0.01)	not MCAR	21.5 (p = 0.011)	not MCAR
MAVIS	11.1 (p = 0.20)	MCAR	19.0 (p = 0.015)	not MCAR
RECORD	108.2 (p < 0.001)	not MCAR	133.8 (p < 0.001)	not MCAR
KAT	91.6 (p < 0.001)	not MCAR	89.0 (p < 0.001)	not MCAR
PRISM	26.9 (p = 0.001)	not MCAR	14.0 (p = 0.12)	MCAR

Table 4 shows the results of the LS test applied to the restricted dataset containing only those patients with a monotone missing data pattern. The majority of patients in MAVIS had monotone missingness with 80% in scenario one and 89% in scenario two. RECORD had only 45% and 69% displaying monotone missingness in scenario one and two respectively. The LS Test generally found evidence against MCAR except for the REFLUX trial, where scenario two was found to be MCAR. As with Little's test, apart from this situation, the conclusion against MCAR occurred for both scenario one and two. Bearing in mind, the LS test is only applicable for monotone missing data, the two methods usually provided the same conclusion; that is, there was evidence against MCAR suggesting missingness was MAR or possibly MNAR.

**Table 4:** Results of the Listing and Schlittgen (LS) test

	Scenario 1			Scenario 2		
LS TEST	N (%)	Test Statistic	In favour of MAR?	N (%)	Test Statistic	In favour of MAR?

REFLUX	287 (80)	2.24 (p = 0.033)	MAR	316 (89)	0.16 (p = 0.39)	not MAR
MAVIS	881 (97)	2.79 (p = 0.008)	MAR	904 (99)	4.02 (p < 0.001)	MAR
RECORD	2401 (45)	10.8 (p < 0.001)	MAR	3634 (69)	12.6 (p < 0.001)	MAR
KAT	1771 (75)	4.45 (p < 0.001)	MAR	1983 (84)	5.23 (p < 0.001)	MAR
PRISM	1103 (83)	7.21 (p < 0.001)	MAR	1118 (84)	5.86 (p < 0.001)	MAR

### Ridout Logistic regression for the missingness mechanism

The first stage was to identify those baseline covariates which were associated with dropout after a particular assessment. All adjusted OR's were less than one implying that those with better QoL at the current assessment were less likely to drop out (data not shown). Table 5 shows the findings from the Ridout logistic regression procedure at each assessment.

**Table 5:** Result of Ridout and Fairclough logistic regression

		**Ridout regression**		**Fairclough logistic regression**	
		Scenario 1	Scenario 2	Scenario 1	Scenario 2
Trial	Assessment	Mechanism	Mechanism	Mechanism	Mechanism
REFLUX	Baseline	MAR	MCAR	-	-
	3 months	MCAR	MCAR	MCAR	MCAR
	12 months	-	-	MAR	MAR

MAVIS	Baseline	MCAR	MCAR	-	-
	6 months	MCAR	MAR	MAR	MAR
	12 months	-	-	MCAR/MAR	MCAR/MAR

RECORD	4 months	MAR	MAR	-	-
	12 months	MAR	MAR	MAR	MAR
	24 months	-	-	MAR	MAR

KAT	Baseline	MCAR	MCAR	-	-
	3 months	MAR	MAR	MAR	MAR
	12 months	MAR	MAR	MAR	MAR
	24 months	-	-	MAR	MAR

PRISM	Baseline	MAR	MAR	-	-
	12 months	MAR	MAR	MAR	MAR
	24 months	-	-	MAR	MAR

RECORD, KAT and PRISM provided consistent conclusions between scenario one and two. Missing data in RECORD and PRISM were found to be MAR, while in KAT data were MCAR at baseline, but MAR at three and 12 months follow up. Some inconsistencies were shown for REFLUX and MAVIS. In REFLUX, ignoring the reminder-response at baseline (scenario one) indicated data were MAR, but including the reminder-response data (scenario two) suggested MCAR. Data were MAR at three months in both scenario one and two. MAVIS data was found to be MCAR at baseline, but scenario one found MCAR data at six months, while scenario two suggested MAR data.

### Fairclough Logistic regression for the missingness mechanism

Firstly the covariates associated with missingness at each assessment were identified and the inclusion of previous QoL was assessed (data not shown). Table 5 shows the findings from Fairclough logistic regression. RECORD and PRISM data were found to be MAR at each assessment for each of the two scenarios. KAT generally displayed MAR except in scenario two where data was MCAR. REFLUX data was found to be MCAR except in scenario one where MAR was found. In MAVIS at six months data were MAR in scenario one but MCAR in scenario two. At 12 months, the inclusion of previous QoL was borderline significant so there was insufficient evidence to conclude MCAR or MAR. Scenario two found the data to be MAR.

### Comparison of immediate and reminder responders using Fairclough's method

In this section, only those responding were considered. The responses received via reminders were set to missing. The advantage is that although reminder-responses were regarded as missing, the actual QoL score was known. Using this approach there was no indication of MNAR for REFLUX, MAVIS and PRISM. In RECORD and KAT however, there was some indication that reminder-response was MNAR since the QoL observed at the particular assessment was found to be a predictor of missingness (reminder-response). Therefore with the assumption that reminder responders are similar to the non-responders, perhaps non-response was also MNAR. This however cannot ever be tested as the data required are missing.

## Discussion

All four methods gave reasonably consistent conclusions for the missingness mechanism within a trial. The two hypothesis tests gave an idea of the global mechanism, while the two logistic regression procedures looked specifically at a particular assessment. The choice between which method should be used should be determined by what is of interest. If the overall mechanism of missing data is of interest then Little's test should be used. This is because this global hypothesis test allows for both monotone and intermittent missing data while the LS test requires monotone missingness. Any inconsistencies between the two methods were mainly due to the fact that the LS test used a subset of the data as not all patients showed a monotone missing data pattern.

If the missing data mechanism at a particular assessment is of interest then either Fairclough's method or Ridout logistic regression can be used. The choice between the two is dependent on which binary indicator is of most relevance. Fairclough distinguishes between missing or not at a particular assessment. Ridout takes responders at a particular assessment and investigates whether they continue and provide a further assessment or whether this is their last assessment and they drop out. Although very similar procedures, the outcome variable is subtly different. The situation that is of most relevance to the researcher drives the choice between the two methods.

The mechanism was not always the same in scenario one and two suggesting the reminder data has an important role to play. In a trial which does not employ a reminder system, only the immediate-responses would be available. If the investigation into the missingness mechanism was based on only this data, then one could potentially get a distorted view. This highlighted that the reminder-responses have an important role to play, not only to increase sample size but to ensure the conclusion on the missing data mechanism is the correct one, to inform the most appropriate analysis strategy. Obtaining as much data as possible is always going to give a more informed decision and ultimately reduce any potential bias in analysis results.

The mechanism of missing data within a particular trial did differ at different assessments using Ridout of Fairclough logistic regression. For example in REFLUX scenario one, there was evidence of MAR after baseline but MCAR after three months using Ridout logistic regression. This difference is likely to be caused by the much smaller amount of missing data and the number of patients with each missing data pattern and particularly the number dropping out after the assessment. At three months of those who provided the assessment (N = 302) only 12 dropped out and thus possibly one reason why there was no evidence against the MCAR assumption. In the larger trials the mechanism of missing data was much more consistent across assessments.

In three of the five trials there was evidence against MCAR data. The advantage of Little's test over the LS test is that it can be applied under any missing data pattern, not just monotone. Intermittent missingness occurred in all five trials and therefore the results of Little's test are more reliable. For two trials, current QoL was impacting on reminder-response and thus there was potentially MNAR data. Usually this conclusion is not possible, and MAR cannot be distinguished from MNAR, as the data required are missing.

It is possible that once patients know they will receive a reminder they may delay response until the reminder is received. The participants would probably not know this until they received their first reminder but at subsequent assessment it would be known. Conversely, once it is known reminders will be sent, this may prompt participants to respond early to avoid being sent the reminder. It was not possible to distinguish the reasons for repeated reminder response or not and it may be part of the participants personality. Some may just be slow-responders and need the reminder to prompt response. In the trials used here the proportion of participants who repeatedly responded by reminder is minimal. In the trials used here the 'learning-effect' of reminders did not appear to be a factor, but it would be interesting to investigate this in future work, as some would argue that only an unexpected reminder is close to the missing data situation.

The sensitivity of different analyses depends on the proportion of missing assessments and the strength of the underlying causes for missing data [18]. In general the undesirable effect of missingness on bias and power increases with the severity of non-randomness as well as the proportion of missingness [19]. It is crucial to identify the mechanism of missingness and thus the most appropriate method for valid analysis and minimum biased results. In the unlikely situation that data can be confirmed as being MCAR, complete case analysis or simple methods of imputation could be used. In the more likely situation of MAR data, multiple imputation is useful [20]. An alternative would be available case analysis and in the longitudinal setting a repeated measures model would be appropriate. When data is thought likely to be MNAR, more sophisticated approaches such as joint modelling or pattern mixtures models should be used [2]. Previously it has been shown that in the presence of MNAR, simple imputation methods were not adequate and perhaps multiple imputation was more suitable [21]. An extension to this work is ongoing where appropriate imputation methods or model-based procedures can be identified for use when the data is known to have a particular mechanism of missingness.

### Strengths and limitations

The main strength of this study was the ability to makes use of reminder data to investigate the missing data mechanism. Previous work has simulated missing data subject to a known mechanism whereas we have used real data to test procedures. The variety of datasets allowed the procedures to be investigated for different proportions of missing data and for different missing data patterns.

Each of the trial datasets employed at least one further QoL measure and the same process as presented above was implemented. Similar findings occurred, suggesting that the results are generalisable to the wider QoL research area and not just to those studies employing the EQ5D measure. The studies themselves were from a wide range of disease areas – surgery for gastro-oesophageal reflux; dietary supplementation for infections in elderly; vitamins and calcium for osteoporosis-related fractures; knee replacement surgery; therapy for Paget's disease. However, these were all trials involving patients with chronic diseases, and the trials used infrequent follow-up (three or more months between assessments). Despite this limitation, we believe that the results should be generalisable to other disease areas, and that the issues surrounding missing data in QoL are the same irrespective of the QoL measure being used. If the data are missing because reduced QoL leads to informative censoring, then this should be taken into consideration in any analysis.

One point to note throughout this work is that data collected via reminder has equal footing to that which was obtained immediately. In the EQ5D instrument the questions refer to health state 'today'. It is possible that filling in questionnaires after reminder may be associated with a certain amount of bias as 'today' has been shifted on in time by a couple of weeks. This is more of an issue if data is being collected at more frequent intervals for example monthly rather than annually, or if it is likely that patients' conditions are changing over the time period because of disease progression or consequences of treatment. In these trials follow up was on at least three or six monthly intervals and therefore this issue was not considered a problem for these studies but would be worth considering in the future.

## Conclusion

We recommend that where possible the reminder data should be collected as it has an important role to play. Records should be kept of which responses were received by reminder and then investigators can make use of the data in the ways we have illustrated. Little's test is applicable for all missing data patterns and therefore is the recommended hypothesis test of MCAR. To obtain a more detailed investigation into the missingness mechanism at a particular assessment, a logistic regression procedure is useful. Deciding between Ridout and Fairclough's approaches would depend on whether the mechanism behind current dropout (Fairclough) or dropout after the assessment in question (Ridout) is of most interest; the choice remains with the researcher.

The methods outlined in this paper are generalisable to any outcome collected by postal questionnaire and not just QoL. The implications for research are that the system of reminders is a useful tool in increasing the response rate of follow-up questionnaires. The data also provide a basis on which an investigation into the missing data mechanism can be undertaken to help inform the most appropriate analysis strategy.

## List of abbreviations

EQ5D: EuroQoL EQ5D health outcome instrument; LS: Listing and Schlittgen; MAR: missing at random; MCAR: missing completely at random; MNAR: missing not at random; QoL: quality of life.

## Competing interests

The authors declare that they have no competing interests.

## Authors' contributions

SF conceived the idea, carried out the analysis and drafted the manuscript. PF and CR supported the analysis and commented on drafts of the manuscript. All authors read and approved the final manuscript.

## Appendix: Detail of the methods to identify the missingness mechanism

### Notation

This section details the notation to be used throughout the description of the missing data mechanism and methods to determine this mechanism. Consider a study with *J *measurements of the outcome (e.g. QoL score). The complete data *Y *is defined as

*Y *= (*y*_*ij*_) where *y*_*ij *_is the value of variable *Y*_*j *_for subject *i*. The matrix *R *defines the pattern of missing data or "missingness" and is defined as *R *= (*r*_*ij*_) where *r*_*ij *_= 0 if *y*_*ij *_is missing and *r*_*ij *_= 1 if *y*_*ij *_is observed. It follows that *R*_*i *_is the vector of indicators of the missing data pattern for the *i*^th ^individual. Let *P *be the number of distinct missing data patterns where *J*^{*p*} ^is the number of observed variables in pattern *p*. The number of cases with the *p*^*th *^pattern is *n*^{*p*} ^and . Let *M*^{*p*} ^be a *J*^{*p*} ^× *J *matrix of indicators of the observed variables in pattern *p*. The matrix has one row for each measure present consisting of (J-1) zero's and one 1 identifying the observed measure. For example, in a study with three assessments where the first and third observation were obtained in the second pattern then



Lastly  is the *J*^{*p*} ^× *1 *vector of means of the observed variables for pattern *p*.

### Mechanism of missingness

The missing data mechanism is described by the conditional distribution of *R *given *Y*, say *f*(*R*|*Y*, *φ*), where *φ *denotes unknown parameters. If missingness does not depend on the values of the data *Y*, missing or observed the data are MCAR; that is



Now let *Y*_*obs *_denote the observed components of *Y *and *Y*_*mis *_the missing components. For MAR, missingness depends only on the observed components of *Y *and not on the missing components, such that



MNAR occurs if the distribution of *R *depends on the missing values in matrix *Y*.

### Little's test [11]

Adapting the description of Fairclough [2] the test statistic arises as follows: the maximum likelihood (ML) estimate of the mean of *Y*_*i *_is  and  is the ML estimate of the covariance of *Y*_*i*_. The ML estimates assume the missing data mechanism is ignorable and are calculated on the available data. It follows that  is the *J*^{*p*} ^× *1 *vector of ML estimates corresponding to the *p*^*th *^pattern and  is the corresponding *J*^{*p*} ^× *J*^{*p*} ^covariance matrix with a correction for degrees of freedom. Little's proposed test statistic when Σ is unknown is,



and is asymptotically chi-squared with (Σ *J*^{*p*} ^- *J*) degrees of freedom [11].

### Listing and Schlittgen test [12]

Some further notation is required for the monotone missing data pattern. Let *w*_*j *_indicate the number of dropouts, at assessment *j*. The observation vectors *y*_*i *_are arranged in a row such that the first *n*_*J *_are observed at all assessments. The next *w*_*J*-1 _vectors *y*_*i *_are observed at all assessments except the last one (i.e. from time 1 to *J-1*). The following *w*_*J*-2 _vectors are observed at *j *= 1, ..., *J*-2 and so on. To construct the overall test statistic the mean of the non-dropouts at a given assessment is based on the first *n*_*J *_observations, leading to



with *n*_*j *_= *n*_*J *_+ *w*_*J*-1 _+ ... + *w*_*J*+1 _for *j*<*J*-1 and *n*_*j *_= *n*_*J *_for *j *= *J*-1.

The statistic  with *w *= *w*_1_+ ... + *w*_*J*-1_.

The statistic *D *takes on large positive (negative) values when all means for the dropouts are smaller (greater) than the ones corresponding to the non-dropouts.

The test statistic  has a normal distribution and , but the variance and correlations must be estimated. The correlations ρ_*kj *_are estimated from the data belonging to the non-dropouts only. The estimation of *σ*^2 ^can be based on the non-dropouts since it is assumed that all *y*_*i *_have the same distribution if the null hypothesis holds.
